# Polycyclic aromatic hydrocarbons and cytochrome P450 in HIV pathogenesis

**DOI:** 10.3389/fmicb.2015.00550

**Published:** 2015-06-02

**Authors:** P. S. S. Rao, Santosh Kumar

**Affiliations:** Department of Pharmaceutical Sciences, College of Pharmacy, University of Tennessee Health Science Center, Memphis, TN, USA

**Keywords:** HIV, smoking, polycyclic aromatic hydrocarbon, cytochrome P450, cancer

## Abstract

High prevalence of cigarette smoking in HIV patients is associated with increased HIV pathogenesis and disease progression. While the effect of smoking on the occurrence of lung cancer has been studied extensively, the association between smoking and HIV pathogenesis is poorly studied. We have recently shown the possible role of cytochrome P450 (CYP) in smoking/nicotine-mediated viral replication. In this review, we focus on the potential role of CYP pathway in polycyclic aromatic hydrocarbons (PAH), important constituents of cigarette smoke, mediated HIV pathogenesis. More specifically, we will discuss the role of CYP1A1 and CYP1B1, which are the major PAH-activating CYP enzymes. Our results have shown that treatment with cigarette smoke condensate (CSC) increases viral replication in HIV-infected macrophages. CSC contains PAH, which are known to be activated by CYP1A1 and CYP1B1 into procarcinogens/toxic metabolites. The expression of these CYPs is regulated by aryl hydrocarbon receptors (AHR), the cellular target of PAH, and an important player in various diseases including cancer. We propose that PAH/AHR-mediated CYP pathway is a novel target to develop new interventions for HIV positive smokers.

## Introduction

According to the world health organization (WHO), human immunodeficiency virus (HIV) is the world’s leading infectious killer with about 40 million reported deaths since early 1980s. Currently, about 35 million people worldwide are estimated to be living with HIV infection or acquired immunodeficiency syndrome (AIDS). As per the center for disease control and prevention (CDC), although the rate of HIV incidence has stabilized in United States, about 50,000 new infections are added to the existing HIV positive population every year.

Tobacco use, cigarette smoking in particular, is highly prevalent amongst HIV-infected populations (Ueda et al., 1989). Several studies have reported 2–3 fold higher prevalence of cigarette smoking in HIV positive patients as compared to general population (Crothers et al., 2009; Tesoriero et al., 2010; Browning et al., 2013). Moreover, numerous independent factors impeding the cessation of cigarette smoking amongst HIV population have been identified. Lower socioeconomic parameters or existing mental illness, for instance, significantly correlates with the rates of cigarette smoking and failure to quit smoking (Shirley et al., 2013). Importantly, concurrent drug use and/or history of substance abuse have been identified as critical factors governing cigarette smoking status in HIV infected individuals (Shirley et al., 2013; Pacek et al., 2014; O’Cleirigh et al., 2015).

In HIV patients, chronic cigarette smoking is known to be a major contributor toward non-AIDS related health issues. Some of the major smoking-mediated complications reported in HIV-infected smokers include increased incidence of pneumonia (Feldman and Anderson, 2013b; Harboe et al., 2014), respiratory infections (Feldman and Anderson, 2013a), low bone mineral density (Kooij et al., 2015), cardiovascular diseases (Smith et al., 2011; Esser et al., 2013), and non-AIDS cancer (Shiels et al., 2010; Smith et al., 2011). In addition to these non-AIDS complications, the most significant deleterious effect of cigarette smoking in HIV smokers is that on HIV pathogenesis (see Impact of Smoking on HIV Pathogenesis).

This review summarizes the findings from literature examining the impact of cigarette smoking on HIV pathogenesis. Specifically, the role of main constituents of cigarette, nicotine and polycyclic aromatic hydrocarbons (PAH), in smoking-mediated increased viral replication has been discussed. Furthermore, the contribution of metabolic enzymes cytochrome P450s (CYPs) toward the observed effects of smoking on HIV replication has been reviewed.

## Smoking and HIV

The predisposition of HIV-infected individuals toward cigarette smoking has resulted in numerous studies examining the effects of cigarette smoking on HIV pathogenesis and vice versa. Moreover, multiple underlying mechanisms governing the effects of smoking on HIV have been reported.

### Impact of HIV Infection on Smoking

Long-term effects of HIV infection are known to cause emergence of the mental health issues including depression (Rueda et al., 2014). Depression is highly prevalent amongst the aging HIV-infected population, which requires a number of treatment modalities including the psychopharmacologic strategies (Benton, 2008). Depression has been found to have significant correlation with enhanced HIV pathogenesis (Rivera-Rivera et al., 2014) and AIDS-related death, especially in women with terminal illness (Cook et al., 2004). Importantly, several studies have reported a close association between depression and cigarette smoking and a heightened inability to quit cigarette smoking by depressed patients (Anda et al., 1990; Breslau et al., 1993; Hayes et al., 2010). For example, in a study comprising 273 subjects the odds of quitting smoking has been found to be relatively lower in the individuals with depressed mood compared with non-depressed individuals. Moreover, medically ill smokers with poor quality of life may need more intensive smoking cessation interventions that includes mood management to help them quit smoking. Therefore, based on the existing literature, it can be rationalized that HIV-associated depression is perhaps a major factor that modulates cigarette smoking habits in HIV-infected patients.

### Impact of Smoking on HIV Pathogenesis

Direct impact of cigarette smoking on HIV replication and progression to AIDS has been examined in several cohort studies. As reviewed by Marshall et al. (2009) various studies have demonstrated a significant relationship between cigarette smoking and rapid decline in CD4 counts, higher risk of developing AIDS, increased risk of acquiring other infections, and enhanced mortality. Among the various observations reported in HIV smokers, the effects of cigarette smoking on plasma viral load in HIV-infected individual is a critical representation for HIV pathogenesis. In seropositive women, for instance, current smoking status significantly correlated with increased plasma viral load (Wojna et al., 2007). Similarly, in a cohort of low-income women, cigarette smoking was reported to have a significant impact on chances of achieving desirable virologic responses (Feldman et al., 2006). A study that examined the effects of smoking among older HIV-positive gay and bisexual individuals reported similar results, wherein cigarette smoking status correlated with lower CD4 counts and higher viral loads (Ompad et al., 2014). Further data analysis in this study revealed that current smokers were significantly less likely to have undetectable plasma viral loads compared to former- and non-smokers. A recent study also reported higher chances of detectable plasma viral load in HIV positive smokers compared to HIV infected non-smokers, which further supports the existing data on cigarette-mediated enhanced HIV pathogenesis (O’Cleirigh et al., 2015). Results from our work corroborates smoking-mediated increased viral replication in HIV-infected smokers compared to HIV positive non-smokers (Ande et al., 2015). Moreover, enhanced p24 levels were observed in HIV-infected macrophages treated with cigarette smoke condensate (CSC) compared to vehicle treated cells thereby providing direct evidence for the impact of cigarette constituents on viral replication in major HIV reservoir. In addition, as reviewed previously, negative impact of smoking on adherence to antiretroviral therapy can be rationalized to worsen the effects of cigarette smoking on HIV pathogenesis (Kumar et al., 2015).

### Smoking-Mediated Enhanced HIV Replication: Implicated Mechanisms

Several cellular mechanisms have been implicated in the smoking-mediated enhanced viral replication. A study that examined the *in vitro* effects of tobacco smoke extract (TSE), for instance, reported enhanced viral replication in TSE treated cells as compared to vehicle-treated control cells (Zhao et al., 2010). Importantly, TSE treatment of Human Jurkat T-cells was accompanied by significant alterations in expression of several genes. Specifically, expression of genes that facilitate HIV infectivity were observed to be upregulated, while genes regulating cellular redox function were significantly downregulated in TSE treated cells. Overall, these changes were rationalized to facilitate the enhanced HIV replication associated with TSE treatment.

Increased cellular oxidative stress and/or diminished antioxidant capacity are known inducers of HIV replications. An early study examined the effects of reactive oxygen species (ROS) in enhancing HIV replication demonstrated the activation of NFκB under increased oxidative stress (Schreck et al., 1991). The critical role played by superoxide anions in propagating HIV replication in human macrophages was later confirmed using a synthetic peroxynitrite decomposition catalyst, MnTBAP (Aquaro et al., 2007). Removal of peroxynitrite resulted in significant reduction in viral replication, in addition to inhibition of lipid peroxidation. Similar interaction between oxidative stress and HIV replication has been observed in clinical samples. For example, compared to HIV-infected patients, in HIV/HCV co-infected patients, higher HIV replication was observed in association with diminished antioxidant capacity to counter the oxidative stress (Shin et al., 2012). Likewise, daily supplements of vitamins C and E to HIV patients resulted in reduced levels of markers for oxidative stress and a trend toward decreased viral load (Allard et al., 1998). Since cigarette smoke is a well-documented inducer of oxidative stress (van der Vaart et al., 2004; Aseervatham et al., 2013), compared to HIV-infected non-smokers, increased HIV replication can be rationalized in HIV positive smokers.

In addition to examining smoking-induced increased oxidative stress, our previous works have investigated the possible involvement of CYPs in regulating smoking-mediated enhanced HIV replication (For review, see Ande et al., 2013). Nicotine-mediated increased production of ROS was confirmed in both U937 cells and SVGA astrocytes (Ande et al., 2012; Jin et al., 2012). In monocytes/macrophages, a major cellular target and reservoir for HIV (Crowe et al., 2003; Jusino, 2014), we have reported a significant role of CYP isoform 2A6 in metabolism of major cigarette constituent, nicotine (Jin et al., 2012). Importantly, in human monocytic U937 cells, CYP2A6-mediated metabolism of nicotine and its metabolite was found to be primarily responsible for generation of procarcinogenic substrates including nicotine derived nitrosamine ketone (NNK; Jin et al., 2012). Similarly, in human astrocytes, the cellular target implicated in HIV-induced neuroAIDS (Eugenin et al., 2011; Henderson et al., 2012), CYP isoforms 1A1 and 2A6 were found to be significantly upregulated following treatment with nicotine (Ande et al., 2012). Moreover, inhibition of the CYP2A6 isoform was associated with significant attenuation of nicotine metabolism. The influence of cigarette smoking on CYP induction was further confirmed in a study wherein significantly increased nicotine metabolism has been observed in HIV-smokers as compared to HIV-positive non-smokers (Earla et al., 2014). A recent report by our group confirmed enhanced oxidative stress in monocytes, as indicated by increased DNA damage, from HIV positive smokers compared to non-smokers (Ande et al., 2015). This increase in oxidative stress was associated with unchanged levels of major antioxidant enzymes and enhanced expression of CYPs. Overall, in addition to increased ROS production, these studies have suggested a strong interaction between nicotine and CYPs isoforms, which may contribute toward enhanced HIV replication in HIV positive smokers.

## Polycyclic Aromatic Hydrocarbons and Cytochrome P450s

**FIGURE 1:**
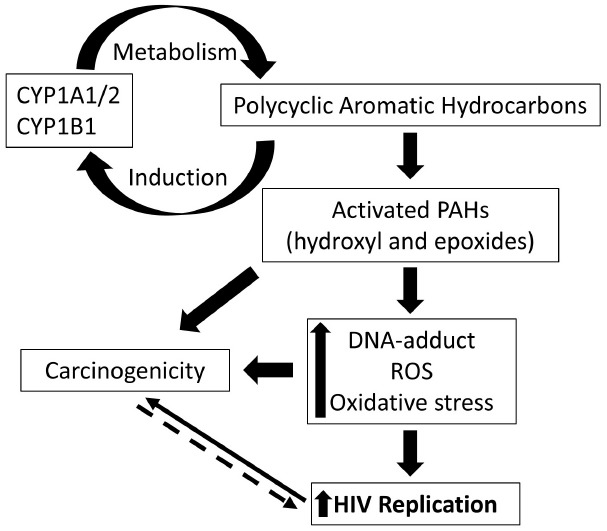
**Schematic representation of the cytochrome P450 (CYP)-mediated effects of polycyclic aromatic hydrocarbons (PAHs) on HIV pathogenesis.** PAHs are known inducers of CYP1A1/1A2/1B1 and these CYP enzymes are responsible for metabolism and activation of PAHs. Activated PAHs and oxidized metabolites mediate increased production of reactive oxygen species, oxidative stress, and DNA-adduct formation. These cellular changes, in turn, are known to enhance HIV replication and mediate carcinogenicity. Increased ROS production, on the other hand, in tumor cells due to mitochondrial dysfunctioning, for instance, can be rationalized to facilitate HIV replication. Targeting the overexpressed CYP 1 enzymes, therefore, serves as a rational clinical approach to impact the incidence of cancer and viral replication in HIV positive smokers.

In addition to the main psychoactive ingredient nicotine, cigarette smoke is known to contain hundreds of (PAHs; Snook et al., 1975). This class of non-polar compounds is known to mediate some of the toxic effects associated with cigarette smoke. Following exposure, several enzymes are responsible for the metabolism and detoxification of PAHs (Shimada, 2006). Importantly, as outlined in Figure 1, bioactivation of PAH by CYP enzymes, isoform 1, have been identified critical in rendering toxicity to these compounds (Burczynski et al., 1999). Moreover, cigarette smoke and PAHs are known to induce expression of CYP1 enzymes via activation of aryl hydrocarbon receptor (AHR)-regulated pathway (Nebert et al., 2004), thereby sustaining the PAH-mediated toxicity. Activation of AHR is followed by nuclear translocation and complex formation with AHR nuclear translocator (ARNT), which results in enhanced expression of CYP enzymes, in particular the isoforms 1A1, 1A2, and 1B1 (Kawajiri and Fujii-Kuriyama, 2007).

Polycyclic aromatic hydrocarbons, in addition to other cigarette constituents, have been classified as highly carcinogenic compounds, and are delivered through cigarette smoke. For instance, at least 10 carcinogenic PAHs have been quantified in mainstream cigarette smoke (Ding et al., 2007). Amongst the various PAHs, benzo[a]pyrene (BP) has long been known and studied for its carcinogenicity (Hecht, 1999). CYP1-mediated oxidation followed by bioactivation of BP results in formation of benzo(a)pyrene-7,8-diol-9,10-epoxide, which is responsible for DNA-adduct formation in lung tissue of smokers, an established risk factor for lung tumor (Alexandrov et al., 2010). Moreover, induction of CYP1A1 and CYP1B1 enzymes by BP further potentiates the formation of carcinogenic DNA adducts (Keshava et al., 2005). Importantly, CYP1A1 genotype was found to predispose smokers to higher degree of BP-induced DNA adduct formation (Alexandrov et al., 2002). Several investigations have, in general, found a significant association between CYP1 polymorphisms and incidence of several types of cancers (Bailey et al., 1998; Kiyohara et al., 2002; Thier et al., 2002; Long et al., 2006; Sergentanis and Economopoulos, 2010). Moreover, direct and indirect impact of these specific CYP1 polymorphisms upon the incidence of tobacco-mediated carcinogenicity have been established (Oyama et al., 2006; Choudhury et al., 2015).

In addition to the well-established AHR driven cellular pathway for CYP1A1 induction, new evidence has suggested a p53-dependent induction of CYP1A1 as an alternate mechanism for BP (Wohak et al., 2014). Interestingly, a previous study has also reported the cellular interactions between BP and p53 wherein, treatment with BP was found to increase the mutability of p53 (Hussain et al., 2001). Mutations in this tumor suppressor protein, p53, is a common feature in many cancers (Vaughan et al., 2014) and further elucidates BP-induced carcinogenicity. In addition, PAH-induced production of ROS has also been implicated in generating p53 mutations, which are known to cause cancer (Yu et al., 2002). Recent studies have also delineated the cellular mechanisms propagating BP-mediated cell cycle progression and metastasis of cancer cells (Guo et al., 2015; Wang et al., 2015).

CYP1B1-mediated PAH toxicity has also been reported in several studies. In fact, compared to CYP1A1, higher constitutive expression of CYP1B1 was found in several organs suggesting a critical role in regulating the intensity of organ-specific PAH-toxicity (Heidel et al., 1998; Shimada et al., 2003). Expression of CYP1B1 is known to be induced in lungs of smokers/ex-smokers compared to non-smokers (Kim et al., 2004; Port et al., 2004). Interestingly, an inverse relationship between hepatic levels of CYP1A1 and extent of CYP1B1-mediated PAH toxicity was reported for BP (Galvan et al., 2003). As reviewed by multiple groups, data from existing studies indicated that genetic predisposition to 1B1 polymorphisms can enhance PAH toxicity and chances for subsequent carcinogenic events (Roos and Bolt, 2005; Li et al., 2015). Moreover, overexpression of CYP1B1 has been observed in several types of cancers (McFadyen et al., 2001; Tokizane et al., 2005; Su et al., 2009). In agreement with these analyses, in an earlier study, CYP1B1 null mice were found to attain protection against the carcinogenic effects of prototypic PAH, 7,12-dimethylbenz[a]anthracene (Heidel et al., 2000).

In addition to propagating carcinogenicity, CYP-mediated activation of PAHs has been implicated in atherogenesis, a major vascular event contributing toward cardiovascular mortality associated with cigarette smoking (Ramos and Moorthy, 2005). Furthermore, deleterious effects of PAHs on viral infections has been highlighted in a study that reported BP-induced enhanced replication of human papillomavirus (Alam et al., 2008).

## PAH and CYP1 Enzymes: Role in HIV Pathogenesis

While the impact of smoking on HIV pathogenesis and replication has been investigated (see Smoking and HIV), the relative contribution of PAHs toward smoking-induced enhanced HIV replication has not been studied. Importantly, the effects of AHR-regulated CYP1 enzymes on HIV pathogenesis remains unclear. However, an earlier work has insinuated a direct interaction between expression levels of CYP1 enzymes and HIV replication. For instance, induction of AHR and AHR-regulated CYP1A1 enzymes by 2,3,7,8-Tetrachlorodibenzo-p-dioxin (TCDD) was associated with enhanced activity of HIV RNA-dependent DNA polymerase and increased expression of viral protein in human T-cells (Tsyrlov and Pokrovsky, 1993). A subsequent study revealed TCDD-induced activation of HIV-1 gene expression *via* an oxidative stress-dependent pathway involving CYP1A1 (Yao et al., 1995).

In addition, recent evidence suggests that predisposition to specific variants of CYP1A1 polymorphs is associated with significant reduction in efficacy of antiretroviral therapy in HIV positive patients (Feldman et al., 2009). A possible explanation provided for this observation was the enhanced ability of CYP1A1 to generate DNA adduct forming metabolites that enhance HIV replication. Moreover, the existing literature provides strong evidence for PAH-mediated enhanced ROS production and oxidative stress (Song et al., 2011; Tsuji et al., 2011; Perumal Vijayaraman et al., 2012), known inducers of viral replication (see Smoking-mediated Enhanced HIV Replication: Implicated Mechanisms). Recently, we have observed that CYP1A1 is highly induced in CSC treated-human monocytic cells, a major HIV target and reservoir, via AHR-mediated pathway (Rao et al., 2015). Overall, the existing literature warrants detailed studies on investigation of the effects of cigarette derived PAHs and CYP isoforms induced by cigarette constituents on HIV replication.

## Concluding Remarks

Existing literatures provide ample evidence to support the deteriorating effects of cigarette smoking on HIV infection. While the study of the effects of nicotine has gained preference, the lack of studies focused on determining the role of PAHs in modulating HIV replication is concerning. In addition to upregulating other CYP isoforms, cigarette/tobacco are known to be strong inducers of CYP 1 enzymes. Importantly, CYP family 1 enzymes play a critical role in modulating the toxicity associated with PAHs.

Our recent data on CSC-induced enhanced HIV replication in primary macrophages (Ande et al., 2015) provides compelling evidence supporting the effects of cigarette constituents in promoting HIV pathogenesis. We rationalize the enhanced CYP1 expression following CSC treatment in HIV-infected macrophages (Rao et al., 2015) to be directly responsible for the observed enhancement in HIV replication. Based on the existing literature supporting the deleterious effects of PAHs, current studies have been designed to study the impact of specific PAHs including BP on HIV replication.

The CYP family 1 enzymes represent an important junction for PAH-mediated enhanced risk for cancer and increased HIV replication (Figure 1). Since there is an association between long-term HIV infection and increased prevalence of cancer in these patients (Engels et al., 2006; Yanik et al., 2013), CYP1-mediated activation of PAHs in smokers can be rationalized to impact the life expectancy in HIV positive smokers. Alternatively, a prominent opinion suggests cancer to be a metabolic disease implicating metabolic dysfunction as the underlying mechanism (Seyfried and Shelton, 2010). Since metabolic dysfunction is known to be associated with increased ROS production and oxidative stress, tumorigenicity can be rationalized to facilitate HIV replication. Hence, it is critical to further delineate the underlying mechanisms that govern the smoking/PAH-mediated changes in HIV replication. Targeting the cellular pathways contributing to CYP1-mediated responses are expected to provide insights into molecular mechanisms underlying co-morbidities in HIV-smokers.

### Conflict of Interest Statement

The authors declare that the research was conducted in the absence of any commercial or financial relationships that could be construed as a potential conflict of interest.
